# A mortality study of beryllium workers

**DOI:** 10.1002/cam4.918

**Published:** 2016-10-20

**Authors:** Paolo Boffetta, Tiffani A. Fordyce, Jack S. Mandel

**Affiliations:** ^1^ Tisch Cancer InstituteIcahn School of Medicine at Mount SinaiNew YorkNew York; ^2^ Exponent Inc.OaklandCalifornia

**Keywords:** Beryllium, lung cancer, mortality, retrospective cohort

## Abstract

We aimed at investigating mortality among beryllium‐exposed workers, according to solubility of beryllium and beryllium compounds. We conducted an historical cohort study of 16,115 workers employed during 1925–2008 in 15 facilities, including eight entailing exposure to insoluble beryllium and seven entailing exposure to soluble/mixed beryllium compounds, who were followed up for mortality until 2011. Data were analyzed using indirect standardization and Cox regression modeling. Lung cancer standardized mortality ratio (SMR, national reference rates) was 1.02 (95% confidence interval [CI]: 0.94–1.10) in the whole cohort, 0.88 (95% CI: 0.75–1.03) in the insoluble beryllium subcohort, and 1.09 (95% CI: 0.99–1.09) in the soluble/mixed beryllium subcohort. For lung cancer, there was an association with period of hire in soluble/mixed beryllium plants but not in insoluble plants, and, conversely, employment in soluble/mixed plants was associated with increased mortality only among workers hired before 1955. There was no trend with duration of employment. Mortality from chronic beryllium disease increased, in particular, among workers hired before 1955 in soluble/mixed beryllium facilities. There was no increase in lung cancer mortality in the entire cohort and lung cancer mortality was not increased among beryllium workers hired in 1955 or later in soluble/mixed beryllium facilities, or at any time among those employed in insoluble beryllium facilities.

## Introduction

An increased mortality from lung cancer was originally reported in workers from two beryllium plants in the United States (Lorain and Reading), who were first employed before 1955 and who were exposed to high levels of beryllium, including soluble beryllium compounds 1, 2. These and an additional five plants were included in a study of 9225 workers employed 1940 through 1969 and followed through 1988 3. In this expanded study mortality from lung cancer was not increased outside the Lorain and Reading plants. Subsequently, a nested case–control study was conducted in the Reading plant 4. In the case–control study, a job‐exposure matrix including quantitative estimates of beryllium exposure was developed 5 and individual exposure indices were correlated with lung cancer mortality. Lung cancer mortality was associated with average and maximum beryllium exposure, only after applying a 10‐ or a 20‐year lag 4. The cohort study was updated to 2005 using 9199 workers 6, 7: the standardized mortality ratio (SMR) for lung cancer, based on national reference rates, was 1.17 (95% confidence interval [CI]: 1.08–1.26) 6; in the 1999–2005 update, there was no excess lung cancer mortality, and again increased mortality was found to be restricted to the Lorain and Reading plants 6. Several indices of beryllium exposure were analyzed in Reading and two other plants with exposure estimates, using different statistical models 6, 7; average and maximum exposure were reportedly associated with lung cancer risk.

Results from experimental studies, as required by European Union legislation, along with the commercial use of beryllium being confined to insoluble forms pointed toward the need to distinguish between beryllium metal and soluble beryllium compounds 8, 9. Soluble compounds are not on the market, and a recent cohort of workers employed in four beryllium facilities with exposure restricted to beryllium metal and insoluble compounds provided no evidence of an increased risk of lung cancer 10. To clarify whether workers are at increased risk of lung cancer, we looked at workers first employed in the beryllium industry under modern exposure circumstances using the year 1955 to distinguish between an “early” and a “modern” period after which initial controls for acute beryllium disease due to exposure to soluble beryllium had been well recognized and a technological change had been adopted to significantly reduce unwanted soluble beryllium in beryllium metal. We then looked at workers exposed only to insoluble beryllium. We conducted an expanded retrospective cohort study of 16,115 workers employed from 1925 to 2008 at 15 U.S. beryllium processing facilities with follow‐up through 2011. This cohort comprised essentially all workers included in previous mortality investigations 3, 6, 10, and included 6890 workers who were employed in facilities which had not been previously studied or had an employment history outside the inclusion period of previous studies.

## Methods

### Cohort enumeration

We assembled a historical cohort of workers employed for at least 1 day in 15 US beryllium manufacturing and distribution facilities which were in operation at some point between 1925 and 2009 (Table S1). Some of the facilities were included in previous cohorts 3, 6, 10. While we know that the overlap of four insoluble beryllium facilities with a previous study 10 is complete, we were not able to estimate the precise overlap with another study of seven facilities because of insufficient information on the members of that cohort 3, 6. The 15 facilities were divided into two groups, based on the chemical and toxicological characteristics of the type of beryllium compounds used: (1) those that only processed insoluble forms of beryllium, including metallic beryllium, beryllium‐containing alloys (primarily copper beryllium), and beryllium oxide (*N *= 8, including the distribution centers and the plant in Reading post‐1965), and (2) those that processed soluble forms of beryllium, including beryllium chloride, fluoride, nitrate, phosphate, and sulfate (tetrahydrate), alone or in combination with insoluble forms (*N* = 7; including the plant in Reading before 1965). The distinction with regard to Reading was made because beryllium ore refining, which uses soluble beryllium compounds, was discontinued at this facility in 1965. Numerous sources of data were used to enumerate the cohort, including employment records and payroll information. Consistency checks were performed on the database to confirm the accuracy of the cohort information.

Each cohort member contributed person‐years of observation from date of first hire until end of follow‐up, defined as date of death or the end of follow‐up (December 31, 2011). For workers with unknown vital status, end of follow‐up was defined as 1 year after their termination date. Missing dates of birth (*N* = 456) were calculated using Social Security numbers as described by Block and colleagues 11. We tested the Block et al. method on a 5% sample of workers with a known date of birth and 97% of the calculated birthdates were within 5 years of the actual birth date. Duration of employment was calculated by subtracting the date of last employment (termination) from the date of first hire. Missing hire dates (*N* = 352) were either assumed to be (1) the date of the first known job, or (2) for employees with no detailed work history information, assumed to be the first date the plant was operational, or (3) if no detailed work history information was available, but there was a termination date, the date was modeled based on average age at hire and length of employment for the particular plant. Missing termination dates (*N* = 551, of whom 331 also had missing hire date) were considered the earlier of either the last date that the plant was operational, the end of the study, or the date of death, in cases with missing hire date. If date of hire was available, the date of termination was modeled based on average age at hire and length of employment for the particular plant. A total of 158 workers (<1%) who were missing multiple data fields essential for the analysis (and who could not be assumed as outlined above) were excluded from the analyses. Workers employed in more than one facility were included in the analyses for all facilities combined for their entire duration of employment, and in the analysis of individual facilities for the length of employment in each particular facility. Employees who worked in more than one facility were included on the soluble/mixed and insoluble analyses only when facilities processed the same type of beryllium, otherwise they were excluded from the soluble/mixed and insoluble analyses, but included in the total cohort.

In the analysis by duration of exposure, continuous employment was assumed. Sensitivity analyses were conducted with lagged exposure of both 10 and 20 years, to account for latency in diseases of interest. Analyses were also conducted according to time since hire and period of hire (before 1955; 1955 or later), the latter variable being a surrogate for technological phases within the industry. In sensitivity analyses, workers employed for <2 days were excluded, since these workers might have been screened out during medical examinations or did not work in the facility after being accepted for employment. Sensitivity analyses were also conducted excluding all workers with missing hire and termination dates, excluding workers who worked less than 2 days (because some cohort members were present only for a medical examination during a single day), <1 year, <5 years, and censoring follow‐up at the age of 85.

### Follow‐up

Vital status was ascertained through December 31, 2011 using data from the National Center for Health Statistics' National Death Index (NDI). The NDI is considered a complete and accurate source of mortality information for US citizens whose death has occurred after January 1, 1979. Cause‐of‐death information coding for individuals identified in the NDI database was obtained through NDI Plus. Workers not identified in the NDI databases who were still employed after 1979 were presumed to still be alive as of the end of study date.

Vital status prior to 1979 was ascertained through the Social Security Administration (SSA), the Pension Benefit Administration Death Audit Service, and various genealogy websites. For suspected and known deaths that occurred prior to 1979, information on the State where the death occurred was obtained and death certificates were requested from the appropriate States. A copy of the verified death certificate was sent to a professional nosologist for coding to the International Classification of Diseases (ICD) revision in effect at the time of death. Only the underlying cause of death was included in the analysis. A total of 292 workers who were not located through NDI, or the SSA, had no other vital status information available in the company records, and were born before January 1, 1912 were considered dead with unknown cause of death, as they were deemed too old to be alive at the end of the study. The date of death was assigned as the later of the date of termination of employment or the date at last contact. An additional 90 workers born after January 1, 1912 who were not located were considered lost to follow‐up as of their date of termination.

### Statistical analysis

Standardized mortality ratios (SMRs) were calculated as ratios of observed to expected deaths. Expected deaths were derived by applying mortality rates from the reference population to calendar period‐, sex‐, and age‐specific categories of person‐years 12. In the main analysis, the national US population was used as reference; sensitivity analyses were conducted by using the population of the facility's State as a reference (Table S1). State rates were not used for all analyses, because with the exception of national rates, The National Center for Health Statistics (NCHS) now requires suppression of causes of death counts with <10 events. When examined by calendar year and age group, there were too many cells that would be suppressed in certain subanalyses (e.g., berylliosis, mesothelioma), which could result in over‐ or underestimation of SMRs. The state rates used for the main analyses of the soluble/mixed and insoluble beryllium subcohorts were obtained before this change went into effect and no rates were suppressed. Observed person‐years and deaths of workers with unknown race (74%) were proportionally allocated based on the proportion of employees in the cohort with known race, and all analyses were weighted by race. The Occupational Mortality Analysis Program, (OCMAP)‐PLUS, was used for calculating SMRs and their 95% confidence intervals (CIs) 13. Heterogeneity between SMRs and linear trends of SMRs were tested according to Breslow and Day 12.

Proportional hazard Cox regression models were fitted to provide hazard ratios (HRs) after adjustment for age and the potential reciprocal confounding effect of the exposure variables. For mortality from all causes, lung cancer and ONMRD, we used life tables to test the proportional hazards assumption, and fitted models included sex, duration of employment, age at hire, period of hire, and type of beryllium plant. Duration of employment was analyzed both as dichotomous variable (<1 year vs. 1 of more years) and as a continuous variable. Similarly, period of hire was analyzed both as dichotomous variable (before 1955 vs. 1955 or late), as a categorical variable (5‐year intervals), and a continuous variable. Interaction terms between period of hire and type of beryllium plants were found statistically significant at *α *= 0.05 and included in additional models. Race was excluded from the models as the majority of records were missing this information. The package SAS 9.4 14 was used for this analysis.

## Results

The cohort comprised a total of 16,115 workers employed over an 84 year period who contributed 567,964 person‐years of observation (Table S2). There were 5,883 workers employed in insoluble beryllium facilities (183,905 person‐years) and 9630 workers employed in soluble/mixed beryllium facilities (356,641 person‐years) (602 workers were employed in both types of facilities and were excluded from the comparative analysis; in a sensitivity analysis, they were included in the soluble/mixed subcohort). The results by main causes of death are reported in Table 1. Overall, workers in the cohort experienced a total of 7,868 deaths vs. 7878.1 expected (SMR: 1.00; 95% CI: 0.98–1.02). The SMR for lung cancer was 1.02 (95% CI: 0.94–1.10); for bronchitis, emphysema, and asthma (COPD, chronic obstructive pulmonary disease), the SMR was 1.00 (95% CI: 0.88–1.13, based on 259 deaths). The only cause of death with a significantly increased SMR was the category “other non‐malignant respiratory diseases” (ONMRD), with 318 observed and 246.8 expected deaths (SMR: 1.29; 95% CI: 1.15–1.44). Details on the causes of death classified as ONMRD are shown in Supplementary Table 3: the category includes 69 deaths from berylliosis, 34 deaths from other lung diseases due to external agents, and 36 deaths from interstitial lung diseases: the latter two groups may include deaths from berylliosis, which justifies the use of the broader category ONMRD in the mortality analysis. A decreased SMR was noticed for several causes, including all malignant neoplasms, liver cancer, leukemia, cerebrovascular diseases, ischemic heart disease, liver cirrhosis, rheumatic heart disease, influenza and pneumonia, and all external causes. Among the causes of death not listed in Table 3, there were 13 deaths from uterine cancer (10.9 expected; SMR: 1.19; 95% CI: 0.64–2.04) and 9 deaths from bone cancer (5.1 expected; SMR: 1.78; 95% CI: 0.81–3.37). Seven of the nine bone cancer deaths occurred in the soluble/mixed subcohort (SMR: 2.05; 95% CI: 0.82– 4.21) and two in the insoluble subcohort (SMR: 1.73; 95% CI: 0.21–6.26). Detailed SMR results according to year of hire, duration of employment, and time since hire are reported in Tables S4 (lung cancer) and 5 (ONMRD); detailed results for other causes of death are available upon request from the authors.

**Table 1:** Standardized mortality ratios for selected causes of death^a^

Cause of Death	Soluble/mixed beryllium				Insoluble beryllium				Total cohort			
	Obs.	Exp.	SMR	95% CI	Obs.	Exp.	SMR	95% CI	Obs.	Exp.	SMR	95% CI
All causes of death	5600	5323.2	1.05^c^	1.03–1.08	1854	2067.1	0.90^c^	0.86–0.94	7868	7878.1	1.00	0.98–1.02
All malignant neoplasms	1298	1380.1	0.94^b^	0.89–0.99	512	546.3	0.94	0.86–1.02	1914	2051	0.93^c^	0.89–0.98
Cancer of oral cavity & pharynx	30	28.7	1.05	0.71–1.49	10	11	0.91	0.44–1,67	42	42.5	0.99	0.71–1.34
Cancer of esophagus	34	36.5	0.93	0.65–1.30	16	15.5	1.04	0.59–1.68	53	55.7	0.95	0.71–1.25
Cancer of stomach	38	45.7	0.83	0.59–1.14	14	14.9	0.94	0.51–1.58	55	64.1	0.86	0.65–1.12
Cancer of colon	119	118.1	1.01	0.84–1.21	49	43.9	1.12	0.83–1.47	177	172.5	1.03	0.88–1.19
Cancer of rectum	18	26.4	0.68	0.40–1.08	10	8.9	1.13	0.54–2.08	32	37.3	0.86	0.59–1.21
Cancer of biliary passages & liver	18	33.6	0.54^c^	0.32–0.85	10	14.9	0.67	0.32–1.24	32	51.7	0.62^c^	0.42–0.87
Cancer of pancreas	58	71.2	0.82	0.62–1.05	24	28.4	0..85	0.54–1.26	87	105.9	0.82	0.66–1.01
Cancer of larynx	14	14.9	0.94	0.51–1.57	3	5.7	0.53	0.11–1.55	19	22.1	0.86	0.52–1.34
Cancer of bronchus, trachea, lung	447	439.0	1.09	0.99–1.19	157	177.9	0.88	0.75–1.03	672	659.9	1.02	0.94–1.10
Cancer of breast	15	25.1	0.60^b^	0.34–0.99	17	15.1	1.13	0.66–1.80	33	40.9	0.81	0.56–1.13
Cancer of prostate (Males only)	104	122.7	0.85	0.69–1.03	46	40.9	1.12	0.82–1.50	158	175.5	0.90	0.77–1.05
Cancer of kidney	29	33.1	0.88	0.59–1.26	12	13.5	0.89	0.46–1.56	42	49.6	0.85	0.61–1.15
Cancer of bladder and other urinary organs	47	41.5	1.13	0.83–1.51	13	14.4	0.90	0.48–1.54	62	59.5	1.04	0.80–1.34
Malignant melanoma of skin	14	20.5	0.68	0.37–1.15	10	9.3	1.08	0.52–1.99	26	31.4	0.83	0.54–1.21
Mesothelioma	43	40.6	1.06	0.77–1.43	16	15.8	1.01	0.58–1.64	62	60.5	1.03	0.79–1.31
Cancer of central nervous system	20	32.8	0.61^b^	0.37–0.94	12	14.3	0.84	0.43–1.47	37	49.8	0.74	0.52–1.02
Non‐hodgkins lymphoma	50	49.9	1.00	0.74–1.32	21	20.3	1.03	0.64–1.58	74	74.6	0.99	0.78–1.25
Leukemia & aleukemia	39	53.6	0.73^b^	0.52–1.00	14	20.7	0.68	0.37–1.14	55	78.9	0.70^c^	0.53–.0.91
Cancer of all other lymphopoietic tissue	12	24.3	0.49^c^	0.26–0.86	9	9.8	0.92	0.42–1.75	23	36.5	0.63^b^	0.40–0.95
All other malignant neoplasms	114	109.6	1.04	0.86–1.25	39	44.8	0.87	0.62–1.19	158	164.4	0.96	0.82–1.12
Benign neoplasms	16	10.9	1.47	0.84–2.39	3	4	0.76	0.16–2.21	20	15.8	1.26	0.77–1.95
Diabetes mellitus	108	114.7	0.94	0.77–1.13	38	49.3	0.77	0.55–1.06	154	175	0.88	0.75–1.03
Cerebrovascular disease	292	319.9	0.91	0.81–1.02	76	106.9	0.71^c^	0.56–0.89	396	454.4	0.87^c^	0.79–0.96
Rheumatic heart disease	10	19.2	0.52^b^	0.25–0.96	5	6.3	0.79	0.26–1.82	15	27.2	0.55^b^	0.31–0.91
Ischemic heart disease	1418	1449.9	0.98	0.93–1.03	456	494	92.3	0.84–1.01	1972	2073.2	0.95^b^	0.91–0.99
Other myocard. insuff.	58	66.0	0.88	0.67–1.14	28	22.7	1.23	0.82–1.78	93	94.8	0.98	0.79–1.20
Hypertension with heart disease	39	51.3	0.76	0.54–1.04	22	20.4	1.08	0.68–1.63	66	76.8	0.86	0.66–1.09
All other heart disease	303	295.2	1.03	0.91–1.15	83	116.7	0.71^c^	0.57–0.88	418	440.5	0.95	0.86–1.05
Hypertension w/o heart disease	29	27.2	1.07	0.72–1.53	10	10.8	0.93	0.45–1.71	43	40.7	1.06	0.76–1.42
Influenza & pneumonia	101	141.9	0.71^c^	0.58–0.87	31	47.6	0.65^b^	0.44–0.93	146	202.4	0.72^c^	0.61–0.85
Bronchitis, emphysema, asthma	187	173.1	1.08	0.93–1.25	60	70.7	0.85	0.65–1.09	259	258.6	1.00	0.88–1.13
Other non‐malignant respiratory disease	227	169.7	1.34^c^	1.17–152	67	60.8	1.10	0.85–1.40	318	246.8	1.29^c^	1.15–1.44
Ulcer of stomach & duodenum	14	16.8	0.83	0.46–1.40	4	5.3	0.75	0.21–1.93	19	23.6	0.81	0.49–1.26
Cirrhosis of liver	73	89.1	0.82	0.64–1.03	31	40.8	0.76	0.52–1.08	107	138.7	0.77^c^	0.63–0.93
Nephritis & nephrosis	61	64.3	0.95	0.73–1.22	21	25.8	0.81	0.50–1.24	87	96.6	0.90	0.72–1.11
All external causes of death	265	300.0	0.88^c^	0.78–01.00	126	157.2	0.80^b^	0.67–0.95	410	485.5	0.85^c^	0.77–0.93
Motor vehicle accidents	69	80.3	0.86	0.67–1.09	34	43	0.79	0.55–1.10	111	130.2	0.85	0.70–1.03
All other accidents	120	117.0	1.03	0.85–1.23	34	54.9	0.62^c^	0.43–0.87	162	182.6	0.89	0.76–1.04
Suicides	68	73.8	0.92	0.72–1.17	46	38.7	1.19	0.87–1.59	117	118.1	0.99	0.82–1.19
Homicides & other external causes	8	29.0	0.28^c^	0.12–0.54	12	20.6	0.58	0.30–1.02	20	54.6	0.37^c^	0.22–0.57
All other causes of death	605	685.7	0.88^c^	0.81–0.96	208	272.6	0.76^c^	0.66–0.87	849	1019.7	0.83^c^	0.78–0.89

Obs, observed deaths; Exp, expected deaths, based on national reference rates; SMR, standardized mortality ratio; CI, confidence interval.

a Causes with 20+ observed or expected deaths in the total cohort are listed.

b *P* < 0.05.

c *P* < 0.01.

Results for lung cancer and ONMRD by facility are reported in Table 2. The SMR for lung cancer was 1.20 (95% CI: 1.01–1.41) for workers employed in Lorain and 1.15 (95% CI: 1.00–1.31) for workers employed before 1965 in Reading, when soluble compounds were used; results for lung cancer at other facilities are unremarkable.

**Table 2:** Standardized mortality ratio of lung cancer and other non‐malignant respiratory diseases (ONMRD) by facility

Facility	Type	*N* workers	Lung cancer			ONMRD		
			Obs	SMR	95% CI	Obs	SMR	95% CI
Chester	S	236	16	1.18	0.68–1.92	8	1.20	0.52–2.37
Cleveland^a^	I	2638	117	0.81	0.67–0.97	55	1.04	0.78–1.35
Delta	S	494	0	[4.9]^c^	0.0–0.75	2	0.64	0.08–2.32
Elmore	S	3302	74	0.75	0.59–0.94	42	1.46	1.05–1.97
Hazleton	S	704	31	0.82	0.56–1.16	28	1.85	1.23–2.67
Lorain	S	1773	138	1.20	1.01–1.41	59	1.16	0.89–1.50
Luckey	S	701	54	1.05	0.79–1.36	23	1.14	0.72–1.70
Reading^d^	S	3241	222	1.15	1.00–1.31	103	1.05	0.85–1.27
Reading^e^	I	864	20	0.80	0.49–1.23	9	1.24	0.57–2.35
Shoemakersville	I	686	10	0.72	0.34–1.32	4	0.87	0.24–2.22
Tucson	I	1197	6	0.64	0.24–1.39	0	[1.7]^c^	0.0–2.14
Distribution centers^b^	I	594	6	1.10	0.40–2.39	1	0.68	0.02–3.80

Obs, observed deaths; SMR, standardized mortality ratio (reference: State rates); CI, confidence interval; S, soluble/mixed beryllium; I, insoluble beryllium.

a Two plants (Perkins, St. Clair).

b Four distribution centers (Elmhurst, Fairfield, Torrance, Warren).

c Expected deaths in square brackets.

d First hire before 1965.

e First hire in 1965 or later.

Cox regression analyses were completed to examine the relationship between exposure variables and mortality from all causes, lung cancer and ONMRD. Age at hire and period of hire were significantly associated with the three outcomes; in addition, type of beryllium facility was associated with overall mortality and there was a decreasing risk of all‐cause mortality for 1 or more year of employment versus <1  year. (Table 3, model I). An effect of short versus long‐term employment was also suggested for ONMRD.

**Table 3:** Hazard ratio of overall mortality, lung cancer, and other non‐malignant respiratory diseases according to short‐term employment, period of hire, and type of beryllium plant

	Hazard ratio (95% CI)		
	All causes of death	Lung cancer	ONMRD
Model I—No interactions terms^a^			
Sex (male vs. female)	1.57 (1.45–1.69)	2.57 (1.88–3.53)	1.21 (0.86–1.70)
Age at hire (1 year increase)	1.09 (1.09–1.10)	1.07 (1.06–1.08)	1.10 (1.09–1.11)
Short‐term employment (1+ year vs. <1 year)	0.92 (0.88–0.96)	1.01 (0.86–1.19)	1.25 (0.98–1.59)
Period of hire (1955 or later vs. before 1955)	0.65 (0.61–0.68)	0.69 (0.57–0.82)	0.69 (0.53–0.89)
Type of beryllium plant (soluble/mixed vs. insoluble)	1.07 (1.01–1.13)	1.04 (0.86–1.26)	1.26 (0.95–1.67)
Model II—with interactions terms^b^			
Sex (male vs. female)	1.57 (1.46–1.69)	2.58 (1.88–3.54)	1.21 (0.86–1.70)
Age at hire (1 year increase)	1.09 (1.09–1.10)	1.07 (1.06–1.08)	1.10 (1.09–1.11)
Short–term employment (1+ year vs. <1 year)	0.93 (088–0.97)	1.03 (0.87–1.21)	1.27 (0.99–1.61)
Period of hire (1955 or later vs. before 1955), stratified by type of beryllium plant			
Insoluble beryllium plants	0.79 (0.72–0.88)	1.03 (0.72–1.50)	0.93 (0.55–1.57)
Soluble/mixed beryllium plants	0.60 (0.56–0.64)	0.59 (0.48–0.74)	0.63 (0.47–0.84)
Type of beryllium plant (soluble/mixed vs. insoluble), stratified by period of hire			
Hire before 1955	1.25 (1.14–1.36)	1.42 (1.04–1.93)	1.58 (1.00–2.49)
Hire in 1955 or later	0.94 (0.87–1.01)	0.82 (0.64–1.06)	1.06 (0.73–1.55)

ONMRD, other non‐malignant respiratory disease.

a Regression models include terms for sex, age at hire, short‐term employment, period of hire, type of beryllium plant, period of hire, and type of beryllium plant.

b Regression models include terms for sex, age at hire, short‐term employment, period of hire, type of beryllium plant, and interaction between period of hire and type of beryllium plant.

A statistically significant interaction was detected between period of hire and type of facility for overall and lung cancer mortality, and interaction terms were added to the regression models: the results for these two variables represent the product of the HR of the main effect and that of the term of interaction with the other variable, and are interpretable as results of stratified analyses (Table 3, model II). HRs for overall mortality were increased for employment in soluble/mixed beryllium facilities relative to insoluble beryllium facilities, while no difference by facility type was detected in workers hired in 1955 or later. Furthermore, in both types of plants, hire in 1955 or later was associated with a reduced mortality compared to hire before 1955, but the association was stronger in soluble/mixed plants. For lung cancer, there was an association with period of hire in soluble/mixed beryllium plants but not in insoluble plants, and, conversely, employment in soluble/mixed plants was associated with increased mortality only among workers hired before 1955. The results of the interaction analysis for ONMRD mortality showed a similar pattern as those for lung cancer.

When duration of employment was treated as a continuous variable, the HR for one additional year of employment were 0.994 (95% CI: 0.991–0.996) for overall mortality, 0.996 (95% CI: 0.988–1.004) for lung cancer, and 1.014 (95% CI: 1.004–1.024) for ONMRD. Analyses based on period of hire as continuous or categorical variable did not provide additional information (results not shown in detail).

Results of sensitivity analyses restricted to men (Table S6) or white subjects (not shown in detail) were very similar to those of the main analysis reported in Table 1. When State mortality rates were used, SMRs were slightly higher and not significantly different from those based on national rates, and trends were consistent (Table S7). Based on the whole cohort, exclusion of workers with <2 days of employment gave results similar to those reported in Table 1, with no changes to the statistical significance of the results. In particular, the SMR for lung cancer was 1.03 (95% CI: 0.93–1.08) and the SMR for ONMRD was 1.30 (95% CI: 1.16–1.46). Excluding workers who worked less than 1 year reduced the proportion of workers with incomplete information on date of birth (2.0%); the SMRs were similar to those of the full analysis (SMR: 0.97 95% CI: 0.87–1.07 for lung cancer; 1.40 95% CI: 1.21–1.61 for ONMRD; Table S8). A further sensitivity analysis excluding workers with <5 years of employment also provided similar results (SMR: 0.94 95% CI: 0.81–1.08 for lung cancer; 1.70 95% CI: 1.41–2.02 for ONMRD; results not reported in detail). Exclusion of workers with missing hire and termination dates also yielded similar SMRs to those reported in Table 1, with no changes to the statistical significance of the results. The SMRs for lung cancer and ONMRD were 1.02 (95% CI: 0.94–1.10) and 1.30 (95% CI: 1.16–1.46), respectively. Analyses with 10‐ and 20‐year lagged exposure yielded similar results, although the SMRs were somewhat lower. Specifically, the SMRs for lung cancer were 1.00 (95% CI: 0.93–1.08) and 0.95 (95% CI: 0.88–1.03) for the analyses with 10‐ and 20‐year lagged exposure, respectively. The SMR for ONMRD for the 10‐year exposure lagged analysis was 1.28 (95% CI: 1.14–1.43) and 1.25 (95% CI: 1.12–1.40) for the 20‐year exposure lagged analysis. A further sensitivity analysis censoring follow‐up at the age of 85 yielded higher SMRs in general; the SMR for mortality from all‐causes was 1.17 (95% CI: 1.15–1.20); the SMR for bronchitis, emphysema, and asthma was 1.14 (95% CI: 1.00–1.28). Results for lung cancer (SMR: 1.05 95% CI: 0.97–1.13) and ONMRD mortality (SMR: 1.38 95% CI: 1.23–1.54) were consistent with those of the main analysis. Results of a further sensitivity analysis in which the 602 workers employed in both types of facilities were included in the soluble/mixed beryllium subcohort were similar to those of the main analysis (lung cancer SMR: 1.06 [95% CI: 0.97–1.15], and ONMRD SMR: 1.16 [95% CI: 1.02–1.31]). Finally, a sensitivity analysis in the insoluble subcohort to account for workers with breaks in their employment history found no changes in the results (a similar analysis was not possible in the soluble/mixed beryllium subcohort because of the larger proportion of workers with incomplete employment history).

## Discussion

This mortality study is the largest cohort of beryllium workers conducted to date. The results show no increase in mortality from any neoplastic or non‐neoplastic cause except ONMRD. The results support the conclusion that an increased mortality from lung cancer was observed among workers initially employed before 1955 in two soluble/mixed beryllium facilities (Lorain and Reading), but not among beryllium workers at any plant in 1955 or later, or those who worked at facilities that only processed insoluble forms of beryllium materials during any period 15, 16. The lack of an increased risk among workers employed in 1955 or later is supported by the Cox regression analysis as the risk of dying from lung cancer for employees in soluble/mixed facilities hired in 1955 or later was lower than the risk of employees hired before 1955. In a sensitivity analysis in which year of hire was stratified as before 1950 versus 1950 or later (because the plant in Lorain was closed in 1949), the excess lung cancer mortality was restricted to the first period (SMR: 1.25; 95% CI: 1.12–1.41 vs. SMR: 0.85; 95% CI: 0.73–0.99). Whether the excess lung cancer mortality in workers hired before 1955 (or 1950) can be attributed to exposure to soluble beryllium compounds or to other factors (e.g., tobacco smoking, exposure to lung carcinogens, or exposure in other industries) has been discussed in previous reports 3, 6, 9, 17, 18 and reviews 15, 16; given the available evidence, it is unlikely that this question will be answered to certainty. Results on duration of employment, and of analyses including lag or excluding short‐term workers also support the conclusion. The lack of an increased lung cancer risk in workers first employed in 1955 or later, and among workers exposed only to insoluble beryllium, is particularly relevant to risk classifications and hazard assessments, since only metallic and insoluble beryllium products are used in commerce.

We studied the mortality from beryllium disease by looking at the general category of ONMRD, as well as at the more specific category “berylliosis” (ICD‐10 code J63.2), which includes both acute and chronic beryllium disease (CBD). The rationale for studying ONMRD is that some deaths from beryllium disease might have been misclassified as another type of ONMRD diseases. As shown in Table S3, the category ONMRD includes 125 deaths from chronic airways obstruction not otherwise specified, which are likely to dilute the association with CBD. Unfortunately, it was not possible to run SMR analyses excluding this group of deaths. The results of these analyses showed an increase in ONMRD mortality in both soluble/mixed beryllium and insoluble beryllium facilities (in the multivariate analysis, type of facility was not associated with ONMRD mortality, suggesting no difference in risk between the two types, as it would be expected since CBD can occur in both types of facilities), with a trend according to duration of employment and time since hire. As expected, one of the greatest risk of death is associated with berylliosis. Although the ICD category “berylliosis” does not separate acute from chronic beryllium disease, it is plausible to assume that most deaths certified with this code were from CBD. Early reports from beryllium plants, including some of those included in this study, show that deaths from acute beryllium disease did not occur after the late‐1940s 19, 20, 21.

The results on mortality from other causes were not remarkable. Among the causes of death reported to be increased in previous analyses of beryllium workers are COPD 6, 22, 23, 24, kidney cancer and chronic kidney disease 3, 6, 25, and cancer of the central nervous system 6, 26, 27: these findings were not confirmed in the present cohort.

Strengths of the study include the retrospective cohort design, the large size of the cohort and the long latency period, the low proportion of lost to follow‐up, and the ability to separately study the commercially relevant subcohort with exposure only to insoluble forms of beryllium. In particular, although the subcohort exposed to insoluble beryllium was younger than that exposed to soluble/mixed beryllium, there were 2937 workers in the insoluble beryllium subcohort with 30 or more years since hire, and the risk did not increase over time. The upper 95% confidence limit of the lung cancer SMR in this category, in which 92 deaths occurred, was 1.16. In other words, shorter latency is not likely to explain the lack of excess of lung cancer in the insoluble beryllium subcohort.

Limitations of the previous studies and this study include the relatively short duration of employment of many cohort members, the lack of information on job titles and on quantitative exposure to beryllium; the lack of information on jobs outside the beryllium industry (68% of cohort members were employed <5 years in the facilities under study); the lack of information on non‐occupational cancer risk factors, mainly tobacco smoking; and the relatively high proportion of cohort members with missing information on some relevant variables. However, we attempted to address these limitations by conducting a large range of sensitivity analyses which were discussed in previous reviews 15, 16, for example, by restricting to men and to white cohort members, excluding short‐term workers, using State reference rates, censoring cohort members at 85 years of age, and applying a 10‐ or 20‐year lag. The results of the sensitivity analyses were robust to the exclusion of workers most likely affected by these potential sources of bias, particularly in workers with less of 12 months of employment. The effect of factors other than employment in the beryllium industry, such as tobacco smoking and employment in high‐risk industries, was partially offset by the use of State mortality rates as a reference. Furthermore, we could not assess with precision the overlap between our cohort and the previous study including seven plants 3, 6, 10, since the individual records of the former study were not made available by authors of the earlier study.

The possibility that workers with prevalent respiratory disease were selected out of the workforce, leading to bias in the form of “healthy worker effect” 28 has been raised in other studies of beryllium workers 6, based on anecdotal evidence 29. It should be stressed, however, that such phenomenon might result in underestimation of the risk of lung cancer and ONMRD when the cohort as a whole is compared with an external population (results in Table 2 and 3), but it would not easily explain patterns of results based on internal comparisons, such as those reported in Table 3 and in Tables S2 and S3.

As with previous cohort studies 3, 6, 10, detailed work history information was not available for the entire cohort. In this case, information was missing for approximately 16% of the cohort and we were unable to use job type as a surrogate for level of exposure. The use of work history to derive indices of exposure to beryllium and other agents will require a substantial effort for data harmonization and exposure assessment, and the lack of detailed exposure data available at the two oldest plants where the only increase in lung cancer mortality was identified will represent an important limitation. In addition, workers' exposure levels to soluble and insoluble beryllium and other agents were very elevated in the 1940s due to ineffective controls making it challenging to discriminate exposure levels by job or a causative agent in this part of the cohort 30.

The lack of correspondence in the results on mortality from lung cancer and beryllium disease (or ONMRD mortality, which we used as proxy for beryllium disease) does not support the hypothesis that chronic inflammation contributes to beryllium‐related lung carcinogenesis 31. Acute beryllium disease has only been associated with exposure to soluble beryllium compounds and may provide a plausible explanation relative to the role of worker exposure to high concentrations of soluble beryllium compounds in the development of lung cancer. In an analysis of mortality of patients in the Beryllium Disease Registry, Steenland and Ward 32 reported that lung cancer excess was more pronounced among those with acute disease than among those with CBD. In the analysis of the seven‐plant study, Ward and colleagues 3 reported that lung cancer was particularly elevated among workers at the Lorain plant with a history of (primarily) acute beryllium disease. We detected an increased mortality from all causes among workers with <5 years employment in soluble/mixed beryllium facilities (Table S2), which may be due to selection of workers who experienced acute beryllium disease out of the workforce. As discussed above, we think all berylliosis deaths which occurred after 1950 were due to CBD.

In conclusion, this study of beryllium workers provides consistent evidence of no increased risk of lung cancer in workers exposed to insoluble beryllium/materials, and among all beryllium workers employed under modern conditions (i.e., in 1955 or later). This finding supports the conclusions of previous reviews 15, 16 that the increased mortality from lung cancer identified in workers employed in the early technological phase of the industry with high exposure to soluble beryllium forms is not relevant to the risk of workers with exposure to insoluble beryllium or having exposure in this industry under modern circumstances to any form of beryllium. As expected, workers in this cohort experienced an increased mortality from beryllium disease for which the difference between soluble/mixed and insoluble forms of beryllium does not appear to be relevant. In light of the confirmation of differing cancer risks by chemical form, hazard classifications for beryllium should be reconsidered especially since soluble forms of beryllium are not commercially utilized.

## Conflict of Interest

None declared.

## Supporting information


**Table S1.** Selected characteristics of the facilities included in the study.
**Table S2.** Selected characteristics of the cohort.
**Table S3.** Causes of death included in the category ‘other non‐malignant respiratory diseases.
**Table S4.** Standardized mortality ratio for lung cancer by year of hire, duration of employment, and time since hire.
**Table S5.** Standardized mortality ratio for other non‐malignant respiratory diseases (ONMRD) by year of hire, duration of employment, and time since hire.
**Table S6.** Standardized mortality ratios for selected causes of death†, by sex, total cohort.
**Table S7.** Standardized mortality ratios for selected causes of death, State reference rates.
**Table S8.** Standardized mortality ratios for selected causes of death, workers with >1 year of employment.
