# Supplementary material for: A mortality study of beryllium workers
Source: Cancer Med. 2016 Oct 20;5(12):3596–605. doi: 10.1002/cam4.918 (PMC5224864; doi:10.1002/cam4.918)
Supplement: Supplementary file 1 — Table S1. Selected characteristics of the facilities included in the study. Table S2. Selected characteristics of the cohort. Table S3. Causes of death included in the category ‘other non‐malignant respiratory diseases. Table S4. Standardized mortality ratio for lung cancer by year of hire, duration of employment, and time since hire. Table S5. Standardized mortality ratio for other non‐malignant respiratory diseases (ONMRD) by year of hire, duration of employment, and time since hire. Table S6. Standardized mortality ratios for selected causes of death†, by sex, total cohort. Table S7. Standardized mortality ratios for selected causes of death, State reference rates. Table S8. Standardized mortality ratios for selected causes of death, workers with >1 year of employment. [file CAM4-5-3596-s001.docx]

Supplementary Table 1. Selected characteristics of the facilities included in the study

| Facility (Soluble/Insoluble) | Date of first hire | Date of last hire | Mean duration of employment (years) | Main production | State for reference rates |
| --- | --- | --- | --- | --- | --- |
| Chester (S) | 1/1/1925 | 1/2/1947 | 1.62 | Extraction from beryl ore. Manufacture of copper beryllium alloy, beryllium metal, beryllium oxide | Ohio |
| Cleveland (Perkins (I) and St. Clair (I)) †‡§ | 2/27/1946 | 2/11/2008 | 4.65 | Perkins: beryllium metal powder making, powder consolidation, beryllium metal machining  St. Clair: machine shop, metallurgical research and development | Ohio |
| Delta (S) | 1/29/1968 | 6/19/2007 | 7.73 | Extraction of beryl and bertrandite ores to beryllium hydroxide | Utah |
| Elmore (S) † | 1/10/1952 | 1/28/2008 | 10.84 | Extraction from beryl ore. Manufacture of copper beryllium alloy, beryllium metal, beryllium oxide | Ohio |
| Hazleton (S) † | 3/4/1957 | 10/30/1978 | 8.06 | Extraction from beryl ore. Manufacture of beryllium metal, beryllium oxide | Pennsylvania |
| Lorain (S) † | 2/22/1935 | 7/9/1948 | 0.77 | Extraction from beryl ore. Manufacture of copper beryllium alloy, beryllium metal, beryllium oxide | Ohio |
| Luckey (S) † | 1/9/1948 | 11/13/1958 | 1.99 | Extraction from beryl ore. Manufacture of beryllium metal | Ohio |
| Reading (S) * † | 6/15/1930 | 11/5/1964 | 6.37 | Extraction from beryl ore. Manufacture of copper beryllium alloy, beryllium metal, beryllium oxide | Pennsylvania |
| Reading (I) ** †‡ | 1/18/1965 | 8/19/2000 | 9.19 | Manufacture of copper beryllium alloy | Pennsylvania |
| Shoemakersville (I) ‡ | 1/11/1955 | 3/19/2007 | 10.20 | Processing of copper beryllium alloys to produce strip, rod, wire products | Pennsylvania |
| Tucson (I) ‡ | 1/2/1980 | 4/29/2008 | 3.32 | Processing of beryllium oxide powder into ceramic products | Arizona |
| Elmhurst DC (I) | 1/1/1976 | 7/2/2007 | 3.66 | Processing and distribution of copper beryllium strip products | Illinois |
| Fairfield DC (I) | 1/25/1968 | 4/25/2005 | 4.67 | Processing and distribution of copper beryllium strip products | New Jersey |
| Torrance DC (I) | 7/15/1968 | 1/4/1999 | 4.43 | Processing and distribution of copper beryllium strip products | California |
| Warren DC (I) | 1/18/1963 | 10/8/2007 | 4.23 | Processing and distribution of copper beryllium rod, bar and tube products | Michigan |

DC, distribution center

S, soluble or mixed soluble/insoluble beryllium

I, insoluble beryllium

* First hire before 1965

** First hire in 1965 or later

† Partial overlap with seven-plant cohort [3-6]

‡ Partial overlap with insoluble beryllium cohort [9]

§ It was not possible to separate workers employed in the two plants in Cleveland

Supplementary Table 2. Selected characteristics of the cohort

|  | Soluble/mixed beryllium facilities | |  | Insoluble beryllium facilities | |  | Total cohort* | |
| --- | --- | --- | --- | --- | --- | --- | --- | --- |
|  | N at risk | Person-years |  | N at risk | Person-years |  | N at risk | Person-years |
| Overall | 9630 | 356640.9 |  | 5883 | 183904.6 |  | 16115 | 567963.7 |
| Gender |  |  |  |  |  |  |  |  |
| Male | 8377 | 311396.7 |  | 4532 | 143276.1 |  | 13492 | 481135.7 |
| Female | 1253 | 45244.1 |  | 1351 | 40628.6 |  | 2623 | 86827.9 |
| Race/ethnicity |  |  |  |  |  |  |  |  |
| White | 9396 | 349579.1 |  | 5580 | 173633.4 |  | 15473 | 543979.3 |
| Non-White | 234 | 7061.8 |  | 303 | 10271.3 |  | 642 | 23984.3 |
| Year of birth |  |  |  |  |  |  |  |  |
| <1920 | 3103 | 101817.8 |  | 637 | 20054.2 |  | 3929 | 129679.2 |
| 1920-1939 | 3821 | 180075.0 |  | 1742 | 72159.5 |  | 5937 | 270328.8 |
| 1940-1959 | 1834 | 60523.7 |  | 2286 | 68569.8 |  | 4156 | 130557.0 |
| 1960+ | 872 | 14224.4 |  | 1218 | 23121.2 |  | 2093 | 37398.6 |
| Year of hire |  |  |  |  |  |  |  |  |
| <1955 | 4902 | 200217.2 |  | 768 | 33398.2 |  | 6004 | 249382.4 |
| 1955+ | 4728 | 156423.7 |  | 5115 | 150506.4 |  | 10111 | 318581.3 |
| Duration of employment |  |  |  |  |  |  |  |  |
| < 5 yrs | 9630 | 270359.8 |  | 5883 | 134582.5 |  | 16115 | 409568.7 |
| 5-14 years | 2805 | 57687.0 |  | 1817 | 34044.9 |  | 5184 | 99322.9 |
| 15-29 yrs | 1203 | 21863.8 |  | 728 | 13197.2 |  | 2415 | 44207.9 |
| 30+ yrs | 403 | 6730.4 |  | 150 | 2080.0 |  | 843 | 14864.2 |
| Time since hire |  |  |  |  |  |  |  |  |
| < 10 yrs | 9630 | 91911.4 |  | 5883 | 57327.6 |  | 16115 | 155258.2 |
| 10-19 years | 8912 | 82669.4 |  | 5520 | 50056.1 |  | 15034 | 138694.2 |
| 20-29 yrs | 7821 | 71896.1 |  | 4542 | 39011.0 |  | 12949 | 116509.4 |
| 30+ yrs | 6493 | 110163.9 |  | 2937 | 37509.9 |  | 9957 | 157501.9 |

* 602 workers employed in both types of facilities were excluded from the main analysis stratified by type of facility (see text for details)

Supplementary Table 3. Causes of death included in the category ‘other non-malignant respiratory diseases.

| Cause of death | N | % |
| --- | --- | --- |
| Berylliosis | 69 | 21.7 |
| Other lung diseases due to external agents | 34 | 10.7 |
| Acute respiratory infections | 0 | 0 |
| Other acute lower respiratory infections | 1 | 0.3 |
| Other diseases of upper respiratory tract | 0 | 0 |
| Bronchiectasis | 3 | 0.9 |
| Suppurative and necrotic conditions of lower respiratory tract | 2 | 0.6 |
| Other diseases of pleura | 4 | 1.3 |
| Other respiratory diseases principally affecting the interstitium | 36 | 11.3 |
| Other diseases of the respiratory system | 31 | 9.7 |
| Chronic airway obstruction, not otherwise classified | 125 | 39.3 |
| Respiratory disease, not otherwise classifiable | 13 | 4.1 |
| Total | 318 | 100.0 |

Supplementary Table 4. Standardized mortality ratio for lung cancer by year of hire, duration of employment and time since hire.

|  | Soluble/mixed beryllium | | | |  | | Insoluble beryllium | | | |  | | Total cohort | | | |
| --- | --- | --- | --- | --- | --- | --- | --- | --- | --- | --- | --- | --- | --- | --- | --- | --- |
|  | Obs | SMR | 95% CI |  | | Obs | | SMR | 95% CI |  | | Obs | | SMR | 95% CI |  |
| Year of hire |  |  |  |  | |  | |  |  |  | |  | |  |  |  |
| < 1955 | 361 | 1.23 | 1.11-1.36 |  | | 45 | | 0.88 | 0.64-1.18 |  | | 428 | | 1.17 | 1.06-1.28 |  |
| 1955+ | 116 | 0.76 | 0.62-0.91 |  | | 112 | | 0.95 | 0.78-1.14 |  | | 244 | | 0.83 | 0.73-0.94 |  |
| p heterogeneity |  | <0.001 |  |  | |  | | 0.31 |  |  | |  | | <0.001 |  |  |
| Duration of employment |  |  |  |  | |  | |  |  |  | |  | |  |  |  |
| < 5 yrs | 371 | 1.11 | 1.00-1.23 |  | | 104 | | 0.91 | 0.74-1.10 |  | | 475 | | 1.05 | 0.96-1.15 |  |
| 5-14 yrs | 64 | 0.97 | 0.74-1.23 |  | | 27 | | 0.84 | 0.55-1.22 |  | | 96 | | 0.92 | 0.74-1.12 |  |
| 15-29 yrs | 29 | 0.92 | 0.62-1.32 |  | | 21 | | 1.18 | 0.73-1.80 |  | | 61 | | 0.95 | 0.73-1.22 |  |
| 30+ yrs | 13 | 0.84 | 0.44-1.43 |  | | 5 | | 1.12 | 0.36-2.61 |  | | 40 | | 1.04 | 0.74-1.41 |  |
| p for linear trend* |  | 0.13 |  |  | |  | | 0.34 |  |  | |  | | 0.58 |  |  |
| Time since hire |  |  |  |  | |  | |  |  |  | |  | |  |  |  |
| < 10 yrs | 2 | 0.14 | 0.02-0.49 |  | | 10 | | 1.03 | 0.49-1.90 |  | | 12 | | 0.48 | 0.25-0.84 |  |
| 10-19 yrs | 30 | 0.85 | 0.57-1.21 |  | | 14 | | 0.61 | 0.34-1.03 |  | | 46 | | 0.75 | 0.55-1.00 |  |
| 20-29 yrs | 72 | 0.99 | 0.78-1.25 |  | | 41 | | 1.03 | 0.74-1.40 |  | | 116 | | 0.96 | 0.79-1.15 |  |
| 30+ yrs | 373 | 1.15 | 1.04-1.28 |  | | 92 | | 0.95 | 0.76-1.16 |  | | 498 | | 1.10 | 1.01-1.20 |  |
| p for linear trend* |  | 0.0004 |  |  | |  | | 0.53 |  |  | |  | | 0.0002 |  |  |

Obs, observed deaths

SMR, standardized mortality ratio (reference: national rates for total cohort; State rates for soluble and insoluble)

CI, confidence interval

* weights are mid-points of categories of duration and time since hire

Supplementary Table 5. Standardized mortality ratio for other non-malignant respiratory diseases (ONMRD) by year of hire, duration of employment and time since hire.

|  | Soluble/mixed beryllium | | |  | Insoluble beryllium | | |  | Total cohort | | |
| --- | --- | --- | --- | --- | --- | --- | --- | --- | --- | --- | --- |
|  | Obs | SMR | 95% CI |  | Obs | SMR | 95% CI |  | Obs | SMR | 95% CI |
| Year of hire |  |  |  |  |  |  |  |  |  |  |  |
| < 1955 | 160 | 1.10 | 0.94-1.29 |  | 21 | 0.98 | 0.60-1.49 |  | 201 | 1.29 | 1.11-1.48 |
| 1955+ | 67 | 1.29 | 1.00-1.64 |  | 46 | 1.26 | 0.93-1.69 |  | 117 | 1.30 | 1.07-1.56 |
| p heterogeneity |  | 0.016 |  |  |  | 0.12 |  |  |  | <0.001 |  |
| Duration of employment |  |  |  |  |  |  |  |  |  |  |  |
| < 5 yrs | 156 | 1.02 | 0.86-1.19 |  | 37 | 0.94 | 0.66-1.29 |  | 194 | 1.12 | 0.96-1.28 |
| 5-14 yrs | 34 | 1.28 | 0.89-1.79 |  | 12 | 1.09 | 0.56-1.91 |  | 48 | 1.34 | 0.99-1.77 |
| 15-29 yrs | 28 | 2.26 | 1.50-3.26 |  | 16 | 2.52 | 1.44-4.10 |  | 51 | 2.16 | 1.61-2.84 |
| 30+ yrs | 9 | 1.94 | 0.89-3.69 |  | 2 | 1.73 | 0.21-6.24 |  | 25 | 1.92 | 1.25-2.84 |
| p for linear trend* |  | <0.001 |  |  |  | 0.003 |  |  |  | <0.001 |  |
| Time since hire |  |  |  |  |  |  |  |  |  |  |  |
| < 10 yrs | 1 | 0.53 | 0.13-2.96 |  | 1 | 0.52 | 0.13-2.88 |  | 2 | 0.55 | 0.06-1.97 |
| 10-19 yrs | 7 | 0.83 | 0.34-1.72 |  | 3 | 0.55 | 0.11-1.60 |  | 10 | 0.81 | 0.39-1.48 |
| 20-29 yrs | 28 | 1.14 | 0.75-1.64 |  | 16 | 1.34 | 0.77-2.18 |  | 46 | 1.40 | 1.01-1.84 |
| 30+ yrs | 191 | 1.18 | 1.02-1.35 |  | 47 | 1.22 | 0.90-1.62 |  | 260 | 1.32 | 1.16-1.49 |
| p for linear trend* |  | 0.30 |  |  |  | 0.24 |  |  |  | 0.06 |  |

Obs, observed deaths

SMR, standardized mortality ratio (reference: national rates for total cohort; State rates for soluble and insoluble)

CI, confidence interval

* weights are mid-points of categories of duration and time since hire

Supplementary Table 6. Standardized mortality ratios for selected causes of death†, by sex, total cohort

|  | Men | | | | Women | | | |
| --- | --- | --- | --- | --- | --- | --- | --- | --- |
| Cause of Death | Obs | Exp | SMR | 95% CI | Obs | Exp | SMR | 95% CI |
| All Causes of Death | 7099 | 7101.8 | 1.00 | 0.98-1.02 | 769 | 776.2 | 0.99 | 0.92-1.06 |
| All Malignant Neoplasms | 1725 | 1839 | 0.94** | 0.89-0.98 | 189 | 212 | 0.89 | 0.77-1.03 |
| Cancer of Oral Cavity & Pharynx | 40 | 40.2 | 1.00 | 0.71-1.36 | 2 | 2.3 | 0.86 | 0.10-3.09 |
| Cancer of Esophagus | 48 | 53.6 | 0.90 | 0.66-1.19 | 5 | 2.1 | 2.40 | 0.78-5.59 |
| Cancer of Stomach | 52 | 59.8 | 0.87 | 0.65-1.14 | 3 | 4.3 | 0.70 | 0.14-2.03 |
| Cancer of Large Intestine | 158 | 153.6 | 1.03 | 0.88-1.20 | 19 | 18.9 | 1.00 | 0.60-1.57 |
| Cancer of Rectum | 30 | 34.1 | 0.88 | 0.59-1.26 | 2 | 3.2 | 0.62 | 0.08-2.23 |
| Cancer of Biliary Passages & Liver | 29 | 47 | 0.62** | 0.41-0.89 | 3 | 4.7 | 0.64 | 0.13-1.87 |
| Cancer of Pancreas | 76 | 94.7 | 0.80 | 0.63-1.01 | 11 | 11.2 | 0.98 | 0.49-1.75 |
| Cancer of Larynx | 18 | 21.5 | 0.84 | 0.50-1.33 | 1 | 0.7 | 1.50 | 0.04-8.34 |
| Cancer of Bronchus, Trachea, Lung | 631 | 612.5 | 1.03 | 0.95-1.11 | 41 | 47.4 | 0.86 | 0.62-1.17 |
| Cancer of Breast | 4 | 2.3 | 1.71 | 0.47-4.39 | 29 | 38.5 | 0.75 | 0.50-1.08 |
| Cancer of Prostate (Males only) | 158 | 175.5 | 0.90 | 0.77-1.05 | n/a | n/a | n/a | n/a |
| Cancer of Kidney | 40 | 46.1 | 0.87 | 0.62-1.18 | 2 | 3.5 | 0.57 | 0.07-2.05 |
| Cancer of Bladder and Other Urinary Organs | 59 | 56.6 | 1.04 | 0.79-1.34 | 3 | 2.8 | 1.06 | 0.22-3.09 |
| Malignant Melanoma of Skin | 24 | 28.9 | 0.83 | 0.53-1.24 | 2 | 2.5 | 0.80 | 0.10-2.91 |
| Mesothelioma | 59 | 55.0 | 1.07 | 0.82-1.38 | 3 | 5.5 | 0.55 | 0.11-1.60 |
| Cancer of Central Nervous System | 32 | 44.7 | 0.72 | 0.49-1.01 | 5 | 5.1 | 0.99 | 0.32-2.31 |
| Non-Hodgkins Lymphoma | 68 | 67 | 1.02 | 0.79-1.29 | 6 | 7.7 | 0.78 | 0.29-1.71 |
| Leukemia & Aleukemia | 50 | 72 | 0.70** | 0.52-0.92 | 5 | 7 | 0.72 | 0.23-1.67 |
| Cancer of All Other Lymphopoietic Tissue | 18 | 32.9 | 0.55** | 0.32-0.87 | 5 | 3.6 | 1.38 | 0.45-3.22 |
| All Other Malignant Neoplasms | 142 | 148 | 0.96 | 0.81-1.13 | 16 | 16.4 | 0.98 | 0.56-1.58 |
| Benign Neoplasms | 20 | 13.9 | 1.44 | 0.88-2.23 | 0 | 2 | --- | 0.00-1.89 |
| Diabetes Mellitus | 139 | 153.3 | 0.91 | 0.76-1.07 | 15 | 21.7 | 0.69 | 0.39-1.14 |
| Cerebrovascular Disease | 350 | 395.5 | 0.89* | 0.80-0.98 | 46 | 58.8 | 0.78 | 0.57-1.04 |
| Rheumatic Heart Disease | 14 | 23.1 | 0.61 | 0.33-1.02 | 1 | 4.1 | 0.24 | 0.01-1.35 |
| Ischemic Heart Disease | 1832 | 1918.8 | 0.96* | 0.91-1.00 | 140 | 154.4 | 0.91 | 0.76-1.07 |
| Other Myocard. Insuff. | 83 | 83 | 1.00 | 0.80-1.24 | 10 | 11.8 | 0.85 | 0.41-1.56 |
| Hypertension with Heart Disease | 54 | 67.3 | 0.80 | 0.60-1.05 | 12 | 9.5 | 1.26 | 0.65-2.20 |
| All Other Heart Disease | 383 | 394.7 | 0.97 | 0.88-1.07 | 35 | 45.7 | 0.77 | 0.53-1.06 |
| Hypertension w/o Heart Disease | 34 | 35 | 0.97 | 0.67-1.36 | 9 | 5.7 | 1.58 | 0.72-3.00 |
| Influenza & Pneumonia | 133 | 181.8 | 0.73** | 0.61-0.87 | 13 | 20.6 | 0.63 | 0.34-1.08 |
| Bronchitis, Emphysema, Asthma | 227 | 230.8 | 0.98 | 0.86-1.12 | 32 | 27.8 | 1.15 | 0.79-1.62 |
| Other Non-malignant Respiratory Disease | 280 | 225.6 | 1.24** | 1.10-1.40 | 38 | 21.2 | 1.79** | 1.27-2.46 |
| Ulcer of Stomach & Duodenum | 17 | 21.7 | 0.78 | 0.46-1.25 | 2 | 1.8 | 1.09 | 0.13-3.95 |
| Cirrhosis of Liver | 95 | 128.7 | 0.74** | 0.60-0.90 | 12 | 10 | 1.20 | 0.62-2.10 |
| Nephritis & Nephrosis | 81 | 86.3 | 0.94 | 74.6-116.7 | 6 | 10.3 | 0.58 | 0.21-1.27 |
| All External Causes of Death | 379 | 453 | 0.84** | 0.76-0.93 | 31 | 32.5 | 0.96 | 0.65-1.36 |
| Motor Vehicle Accidents | 97 | 121.4 | 0.80* | 0.65-0.98 | 14 | 8.8 | 1.58 | 0.87-2.66 |
| All Other Accidents | 153 | 168.7 | 0.91 | 0.77-1.06 | 9 | 13.8 | 0.65 | 0.30-1.24 |
| Suicides | 112 | 111.9 | 1.00 | 0.82-1.20 | 5 | 6.2 | 0.81 | 0.26-1.89 |
| Homicides & Other External Causes | 17 | 51 | 0.33** | 0.19-0.53 | 3 | 3.6 | 0.83 | 0.17-2.41 |
| All Other Causes of Death | 730 | 889 | 0.82** | 0.76-0.88 | 119 | 130.7 | 0.91 | 0.75-1.09 |

† Causes with 20+ observed or expected deaths in total cohort are listed

* p<0.05

** p<0.01

Obs, observed deaths

Exp, expected deaths, based on national reference rates

SMR, standardized mortality ratio

CI, confidence interval

Supplementary Table 7. Standardized mortality ratios for selected causes of death, State reference rates†

|  | Soluble/mixed beryllium | | | | Insoluble beryllium | | | | Total cohort | | | |
| --- | --- | --- | --- | --- | --- | --- | --- | --- | --- | --- | --- | --- |
| Cause of Death | Obs. | Exp. | SMR | 95% CI | Obs. | Exp. | SMR | 95% CI | Obs. | Exp. | SMR | 95% CI |
| All Causes of Death | 5600 | 5548.7 | 1.01 | 0.98- 1.04 | 1854 | 2044.1 | 0.91** | 0.87-0.95 | 7868 | 8009.5 | 0.98 | 0.96-1.00 |
| All Malignant Neoplasms | 1298 | 1434 | 0.91** | 0.86-0.96 | 512 | 544.3 | 0.94 | 0.86-1.03 | 1914 | 2054.2 | 0.93** | 0.89-0.97 |
| Cancer of Oral Cavity & Pharynx | 30 | 28 | 1.07 | 0.72-1.53 | 10 | 10.9 | 0.92 | 0.44-1.68 | 42 | 42.6 | 0.99 | 0.71-1.33 |
| Cancer of Esophagus | 34 | 40.6 | 0.84 | 0.58-1.17 | 16 | 15.9 | 1.00 | 0.57-1.63 | 53 | 57.5 | 0.92 | 0.69-1.21 |
| Cancer of Stomach | 38 | 47.3 | 0.80 | 0.57-1.10 | 14 | 15.9 | 0.88 | 0.48-1.48 | 55 | 69.9 | 0.79 | 0.59-1.03 |
| Cancer of Large Intestine | 119 | 132.1 | 0.90 | 0.75-1.08 | 49 | 45.3 | 1.08 | 0.80-1.43 | 177 | 180.9 | 0.98 | 0.84-1.13 |
| Cancer of Rectum | 18 | 30.2 | 0.60* | 0.35-0.94 | 10 | 9.5 | 1.06 | 0.51-1.94 | 32 | 41.1 | 0.78 | 0.53-1.10 |
| Cancer of Biliary Passages & Liver | 18 | 31.8 | 0.57* | 0.34-0.90 | 10 | 15.2 | 0.66 | 0.32-1.21 | 32 | 53 | 0.60** | 0.41-0.85 |
| Cancer of Pancreas | 58 | 69.8 | 0.83 | 0.63-1.07 | 24 | 28.5 | 0.84 | 0.54-1.25 | 87 | 106.2 | 0.82 | 0.66-1.01 |
| Cancer of Larynx | 14 | 15.8 | 0.88 | 0.48-1.48 | 3 | 5.6 | 0.54 | 0.11-1.57 | 19 | 21.8 | 0.87 | 0.52-1.35 |
| Cancer of Bronchus, Trachea, Lung | 477 | 446.1 | 1.07 | 0.98-1.17 | 157 | 169.8 | 0.93 | 0.79-1.08 | 672 | 625.3 | 1.08 | 99.5-1.16 |
| Cancer of Breast | 15 | 26.7 | 0.56* | 0.32-0.93 | 17 | 15.7 | 1.08 | 0.63-1.73 | 33 | 43 | 0.77 | 0.53-1.08 |
| Cancer of Prostate (Males only) | 104 | 126 | 0.83 | 0.67-1.00 | 46 | 40.8 | 1.13 | 0.83-1.51 | 158 | 180.7 | 0.87 | 0.74-1.02 |
| Cancer of Kidney | 29 | 33.7 | 0.86 | 0.58-1.24 | 12 | 13.4 | 0.90 | 0.46-1.57 | 42 | 49.3 | 0.85 | 0.62-1.15 |
| Cancer of Bladder and Other Urinary Organs | 47 | 45 | 1.04 | 0.77-1.39 | 13 | 15.1 | 0.86 | 0.46-1.47 | 62 | 63.6 | 0.97 | 0.75-1.25 |
| Malignant Melanoma of Skin | 14 | 19.9 | 0.70 | 0.38-1.18 | 10 | 9.2 | 1.09 | 0.52-2.00 | 26 | 30.9 | 0.84 | 0.55-1.23 |
| Cancer of Central Nervous System | 20 | 31.3 | 0.64* | 0.39- 0.99 | 12 | 14 | 0.85 | 0.44-1.49 | 37 | 48.7 | 0.76 | 0.54-1.05 |
| Non-Hodgkins Lymphoma | 50 | 52.7 | 0.95 | 0.70-1.25 | 21 | 21 | 1.00 | 0.62-1.53 | 74 | 77.0 | 0.96 | 0.76-1.21 |
| Leukemia & Aleukemia | 39 | 54.8 | 0.71* | 0.51-0.97 | 14 | 20.8 | 0.67 | 0.37-1.13 | 55 | 80.2 | 0.69** | 0.52-0.89 |
| Cancer of All Other Lymphopoietic Tissue | 12 | 24.8 | 0.48** | 0.25-0.85 | 9 | 9.8 | 0.92 | 0.42-1.75 | 23 | 36.5 | 0.63* | 0.40-0.95 |
| All Other Malignant Neoplasms | 114 | 121.7 | 0.94 | 0.77-1.13 | 39 | 45.2 | 0.86 | 0.61-1.18 | 158 | 166.1 | 0.95 | 0.81-1.11 |
| Benign Neoplasms | 16 | 12.2 | 1.31 | 0.75-2.13 | 3 | 4.1 | 0.74 | 0.15-2.15 | 20 | 16.8 | 1.19 | 0.73-1.84 |
| Diabetes Mellitus | 108 | 134.7 | 0.80* | 0.66-0.97 | 38 | 50.8 | 0.75 | 0.53-1.03 | 154 | 184.0 | 0.84* | 0.71-0.98 |
| Cerebrovascular Disease | 292 | 322.9 | 0.90 | 0.80-1.01 | 76 | 105.4 | 0.72** | 0.57-0.90 | 396 | 471.9 | 0.84** | 0.76-0.93 |
| Rheumatic Heart Disease | 10 | 23.3 | 0.43** | 0.21-0.79 | 5 | 6.9 | 0.72 | 0.24-1.69 | 15 | 30.6 | 0.49** | 0.27-0.81 |
| Ischemic Heart Disease | 1418 | 1529 | 0.93** | 0.88-0.98 | 456 | 501.5 | 0.91* | 0.83-1.00 | 1972 | 2175.7 | 0.91** | 0.87-0.95 |
| Other Myocard. Insuff. | 58 | 74.9 | 0.77 | 0.59-1.00 | 28 | 22.1 | 1.27 | 0.84-1.83 | 93 | 100.9 | 0.92 | 0.74-1.13 |
| Hypertension with Heart Disease | 39 | 50.6 | 0.77 | 0.55-1.05 | 22 | 21.9 | 1.01 | 0.63-1.52 | 66 | 85.0 | 0.78* | 0.60-0.99 |
| All Other Heart Disease | 303 | 323.7 | 0.94 | 0.83-1.05 | 83 | 112.3 | 0.74** | 0.59-0.92 | 418 | 440.6 | 0.95 | 0.86-1.04 |
| Hypertension w/o Heart Disease | 29 | 27 | 1.07 | 0.72-1.54 | 10 | 10.8 | 0.92 | 0.44-1.70 | 43 | 43.0 | 1.00* | 0.73-1.35 |
| Influenza & Pneumonia | 101 | 132.1 | 0.77** | 0.62-0.93 | 31 | 48.2 | 0.64* | 0.44-0.91 | 146 | 221.7 | 0.66** | 0.56-0.78 |
| Bronchitis, Emphysema, Asthma | 187 | 168.2 | 1.11 | 0.96-1.28 | 60 | 68.1 | 0.88 | 0.67-1.13 | 259 | 251.9 | 1.03 | 0.91-1.16 |
| Other Non-malignant Respiratory Disease | 227 | 197.4 | 1.15* | 1.01-1.31 | 67 | 57.9 | 1.16 | 0.90-1.47 | 318 | 246.6 | 1.29** | 1.15-1.44 |
| Ulcer of Stomach & Duodenum | 14 | 15.5 | 0.90 | 0.49-1.51 | 4 | 5.6 | 0.71 | 0.19-1.82 | 19 | 25.7 | 0.74 | 0.44-1.15 |
| Cirrhosis of Liver | 73 | 79.1 | 0.92 | 0.72-1.16 | 31 | 45.3 | 0.68* | 0.47-0.97 | 107 | 153.2 | 0.70** | 0.57-0.84 |
| Nephritis & Nephrosis | 61 | 77.3 | 0.79 | 0.60-1.01 | 21 | 25.3 | 0.83 | 0.51-1.27 | 87 | 96.8 | 0.90 | 0.72-1.11 |
| All External Causes of Death | 265 | 262.9 | 1.01 | 0.89-1.14 | 126 | 144 | 0.88 | 0.73-1.04 | 410 | 443 | 0.92 | 0.84-1.02 |
| Motor Vehicle Accidents | 69 | 66.2 | 1.04 | 0.81-1.32 | 34 | 37.8 | 0.90 | 0.62-1.26 | 111 | 112.5 | 0.99 | 0.81-1.19 |
| All Other Accidents | 120 | 109.8 | 1.09 | 0.91-1.31 | 34 | 51 | 0.67* | 0.46-0.93 | 162 | 170.3 | 0.95 | 0.81-1.11 |
| Suicides | 68 | 67 | 1.02 | 0.79-1.29 | 46 | 36.8 | 1.25 | 0.92-1.67 | 117 | 112.9 | 1.04 | 0.86-1.24 |
| Homicides & Other External Causes | 8 | 20 | 0.40** | 0.17-0.79 | 12 | 18.4 | 0.65 | 0.34-1.14 | 20 | 47.3 | 0.42** | 0.26-0.65 |
| All Other Causes of Death | 605 | 744.6 | 0.81** | 0.75-0.88 | 208 | 260.5 | 0.80** | 0.69-0.92 | 849 | 1016.1 | 0.84** | 0.78-0.89 |

† Causes with 20+ observed or expected deaths for the total cohort are listed

* p<0.05

** p<0.01

Obs, observed deaths

Exp, expected deaths, based on state reference rates

SMR, standardized mortality ratio

CI, confidence interval

Supplementary Table 8. Standardized mortality ratios for selected causes of death, workers with > 1 year of employment

|  | Soluble/mixed beryllium | | | | Insoluble beryllium | | | | Total cohort | | | |
| --- | --- | --- | --- | --- | --- | --- | --- | --- | --- | --- | --- | --- |
| Cause of Death | Obs. | Exp. | SMR | 95% CI | Obs. | Exp. | SMR | 95% CI | Obs. | Exp. | SMR | 95% CI |
| All Causes of Death | 2459 | 2606.4 | 0.94** | 0.91-0.98 | 1172 | 1341.1 | 0.83** | 0.83-0.93 | 4045 | 4345.1 | 0.93** | 0.90-0.96 |
| All Malignant Neoplasms | 590 | 687.2 | 0.86** | 0.79-0.93 | 330 | 359.1 | 0.92 | 0.92-1.02 | 1024 | 1147.3 | 0.89** | 0.84-0.95 |
| Cancer of Oral Cavity & Pharynx | 12 | 13.3 | 0.90 | 0.47-1.57 | 5 | 7.2 | 0.69 | 0.69-1.62 | 19 | 23.7 | 0.80 | 0.48-1.25 |
| Cancer of Esophagus | 18 | 20.3 | 0.89 | 0.53-1.40 | 12 | 10.7 | 1.12 | 1.12-1.96 | 33 | 32.2 | 1.03 | 0.71-1.44 |
| Cancer of Stomach | 17 | 21.7 | 0.79 | 0.46-1.26 | 8 | 10.4 | 0.77 | 0.77-1.52 | 28 | 34.5 | 0.81 | 0.54-1.18 |
| Cancer of Colon | 54 | 62 | 0.87 | 0.65-1.14 | 28 | 29.7 | 0.94 | 0.94-1.36 | 91 | 94.8 | 0.96 | 0.77-1.18 |
| Cancer of Rectum | 5 | 14 | 0.36* | 0.12-0.83 | 2 | 6.2 | 0.32 | 0.32-1.17 | 11 | 20.2 | 0.54* | 0.27-0.97 |
| Cancer of Biliary Passages & Liver | 6 | 15.6 | 0.39* | 0.14-0.84 | 6 | 10.1 | 0.59 | 0.59-1.29 | 16 | 29.8 | 0.54** | 0.31-0.87 |
| Cancer of Pancreas | 24 | 34 | 0.71 | 0.45-1.05 | 14 | 18.9 | 0.74 | 0.74-1.24 | 43 | 59.5 | 0.72* | 0.52-0.97 |
| Cancer of Bronchus, Trachea, Lung | 219 | 217 | 1.01 | 0.88-1.15 | 105 | 112.6 | 0.93 | 0.93-1.13 | 362 | 374.3 | 0.97 | 0.87-1.07 |
| Cancer of Breast | 9 | 11.9 | 0.76 | 0.35-1.44 | 9 | 9.8 | 0.92 | 0.92-1.74 | 19 | 21.3 | 0.89 | 0.54-1.39 |
| Cancer of Prostate (Males only) | 47 | 57 | 0.83 | 0.61-1.10 | 32 | 27 | 1.18 | 1.18-1.67 | 87 | 95.0 | 0.92 | 0.73-1.13 |
| Cancer of Kidney | 13 | 16.7 | 0.78 | 0.42-1.34 | 9 | 8.9 | 1.01 | 1.01-1.92 | 23 | 28.3 | 0.81 | 0.52-1.22 |
| Cancer of Bladder and Other Urinary Organs | 26 | 21.4 | 1.22 | 0.79-1.78 | 8 | 10 | 0.80 | 0.80-1.58 | 36 | 32.8 | 1.10 | 0.77-1.52 |
| Cancer of Central Nervous System | 12 | 15.7 | 0.77 | 0.40-1.34 | 9 | 9.3 | 0.97 | 0.97-1.84 | 26 | 28.6 | 0.91 | 0.59-1.33 |
| Non-Hodgkins Lymphoma | 28 | 25.8 | 1.09 | 0.72-1.57 | 12 | 13.9 | 0.87 | 0.87-1.51 | 43 | 42.2 | 1.02 | 0.74-1.37 |
| Leukemia & Aleukemia | 16 | 26.5 | 0.60* | 0.35-0.98 | 11 | 13.7 | 0.80 | 0.80-1.44 | 29 | 44.1 | 0.66* | 0.44-0.94 |
| Cancer of All Other Lymphopoietic Tissue | 5 | 11.8 | 0.42* | 0.14-0.99 | 8 | 6.5 | 1.24 | 1.24-2.44 | 15 | 20.5 | 0.73 | 0.41-1.21 |
| All Other Malignant Neoplasms | 51 | 59.1 | 0.86 | 0.64-1.14 | 25 | 29.9 | 0.84 | 0.84-1.24 | 81 | 92.8 | 0.87 | 0.69-1.09 |
| Diabetes Mellitus | 54 | 65.1 | 0.83 | 0.62-1.08 | 25 | 33.6 | 0.75 | 0.75-1.10 | 87 | 98.9 | 0.88 | 0.71-1.09 |
| Cerebrovascular Disease | 121 | 144.9 | 0.84* | 0.69-1.00 | 47 | 68.2 | 0.69** | 0.69-0.92 | 196 | 239.7 | 0.82** | 0.71-0.94 |
| Ischemic Heart Disease | 602 | 701.1 | 0.86** | 0.79-0.93 | 284 | 326.8 | 0.87* | 0.87-0.98 | 984 | 1114.3 | 0.88** | 0.83-0.94 |
| Other Myocard. Insuff. | 26 | 33.8 | 0.77 | 0.50-1.13 | 20 | 14.5 | 1.38 | 1.38-2.13 | 53 | 50.5 | 1.05 | 0.79-1.37 |
| Hypertension with Heart Disease | 19 | 22.9 | 0.83 | 0.50-1.30 | 15 | 14.3 | 1.05 | 1.05-1.73 | 39 | 41.7 | 0.94 | 0.67-1.28 |
| All Other Heart Disease | 123 | 151.3 | 0.81* | 0.68-0.97 | 53 | 73.8 | 0.72* | 0.72-0.94 | 208 | 242.6 | 0.86* | 0.75-0.98 |
| Hypertension w/o Heart Disease | 10 | 12.3 | 0.81 | 0.39-1.50 | 8 | 7 | 1.14 | 1.14-2.24 | 22 | 22 | 1.00 | 0.63-1.52 |
| Influenza & Pneumonia | 52 | 59.2 | 0.88 | 0.66-1.15 | 23 | 31.5 | 0.73 | 0.73-1.10 | 89 | 107.2 | 0.83 | 0.67-1.02 |
| Bronchitis, Emphysema, Asthma | 81 | 82.2 | 0.99 | 0.78-1.23 | 36 | 45 | 0.80 | 0.80-1.11 | 129 | 146.3 | 0.88 | 0.74-1.05 |
| Other Non-malignant Respiratory Disease | 115 | 90 | 1.28* | 1.06-1.53 | 48 | 38.2 | 1.26 | 1.26-1.67 | 187 | 133.8 | 1.40** | 1.21-1.61 |
| Cirrhosis of Liver | 35 | 38.8 | 0.90 | 0.63-1.26 | 23 | 30 | 0.77 | 0.77-1.15 | 61 | 79.9 | 0.76* | 0.58-0.98 |
| Nephritis & Nephrosis | 25 | 35.9 | 0.70 | 0.45-1.03 | 8 | 16.6 | 0.48* | 0.48-0.95 | 38 | 53.1 | 0.72* | 0.51-0.98 |
| All External Causes of Death | 124 | 137.4 | 0.90 | 0.75-1.08 | 78 | 94.4 | 0.83 | 0.83-1.03 | 221 | 287.2 | 0.77** | 0.67-0.88 |
| Motor Vehicle Accidents | 34 | 36 | 0.95 | 0.66-1.32 | 21 | 24.7 | 0.85 | 0.85-1.30 | 63 | 78.1 | 0.81 | 0.62-1.03 |
| All Other Accidents | 57 | 54.7 | 1.04 | 0.79-1.35 | 19 | 33.3 | 0.57* | 0.57-0.89 | 84 | 104.8 | 0.80* | 0.64-0.99 |
| Suicides | 29 | 36 | 0.81 | 0.54-1.16 | 29 | 24.2 | 1.20 | 1.20-1.72 | 61 | 70.7 | 0.86 | 0.66-1.11 |
| Homicides & Other External Causes | 4 | 10.7 | 0.37* | 0.10-0.95 | 9 | 12.1 | 0.74 | 0.74-1.41 | 13 | 33.6 | 0.39** | 0.21-0.66 |
| All Other Causes of Death | 273 | 346.6 | 0.79** | 0.70-0.89 | 132 | 170.4 | 0.77** | 0.77-0.92 | 441 | 558.9 | 0.79** | 0.72-0.87 |

* p<0.05

** p<0.01

Obs, observed deaths

Exp, expected deaths, based on national reference rates

SMR, standardized mortality ratio

CI, confidence interval
